# *Brugia malayi* Microfilariae Induce a Regulatory Monocyte/Macrophage Phenotype That Suppresses Innate and Adaptive Immune Responses

**DOI:** 10.1371/journal.pntd.0003206

**Published:** 2014-10-02

**Authors:** Noëlle Louise O'Regan, Svenja Steinfelder, Gopinath Venugopal, Gopala B. Rao, Richard Lucius, Aparna Srikantam, Susanne Hartmann

**Affiliations:** 1 Freie Universität Berlin, Center for Infection Medicine, Institute of Immunology, Berlin, Germany; 2 Blue Peter Public Health and Research Centre-LEPRA Society, Hyderabad, Andhra Pradesh, India; 3 Humboldt Universität Berlin, Department of Biology, Molecular Parasitology, Berlin, Germany; Queensland Institute of Medical Research, Australia

## Abstract

**Background:**

Monocytes and macrophages contribute to the dysfunction of immune responses in human filariasis. During patent infection monocytes encounter microfilariae in the blood, an event that occurs in asymptomatically infected filariasis patients that are immunologically hyporeactive.

**Aim:**

To determine whether blood microfilariae directly act on blood monocytes and *in vitro* generated macrophages to induce a regulatory phenotype that interferes with innate and adaptive responses.

**Methodology and principal findings:**

Monocytes and *in vitro* generated macrophages from filaria non-endemic normal donors were stimulated *in vitro* with *Brugia malayi* microfilarial (Mf) lysate. We could show that monocytes stimulated with Mf lysate develop a defined regulatory phenotype, characterised by expression of the immunoregulatory markers IL-10 and PD-L1. Significantly, this regulatory phenotype was recapitulated in monocytes from *Wuchereria bancrofti* asymptomatically infected patients but not patients with pathology or endemic normals. Monocytes from non-endemic donors stimulated with Mf lysate directly inhibited CD4^+^ T cell proliferation and cytokine production (IFN-γ, IL-13 and IL-10). IFN-γ responses were restored by neutralising IL-10 or PD-1. Furthermore, macrophages stimulated with Mf lysate expressed high levels of IL-10 and had suppressed phagocytic abilities. Finally Mf lysate applied during the differentiation of macrophages *in vitro* interfered with macrophage abilities to respond to subsequent LPS stimulation in a selective manner.

**Conclusions and significance:**

Conclusively, our study demonstrates that Mf lysate stimulation of monocytes from healthy donors *in vitro* induces a regulatory phenotype, characterized by expression of PD-L1 and IL-10. This phenotype is directly reflected in monocytes from filarial patients with asymptomatic infection but not patients with pathology or endemic normals. We suggest that suppression of T cell functions typically seen in lymphatic filariasis is caused by microfilaria-modulated monocytes in an IL-10-dependent manner. Together with suppression of macrophage innate responses, this may contribute to the overall down-regulation of immune responses observed in asymptomatically infected patients.

## Author Summary

Lymphatic filariasis is a parasitic disease that affects over one million people worldwide, causing chronic morbidity in the majority of infected individuals. A certain proportion of individuals develop asymptomatic infection that allows persistence of the parasite and therefore transmission of disease through the blood-circulating microfilariae. We show that monocytes and macrophages, innate effector cells that circulate in the blood, migrate or reside in tissues and come into contact with microfilariae, can be stimulated by microfilarial (Mf) lysate *in vitro* to develop a regulatory phenotype via expression of PD-L1 and IL-10. Significantly, this regulatory monocyte phenotype was directly reflected in monocytes isolated from filaria asymptomatically infected patients in the absence of external stimuli. Mf lysate-modulated monocytes inhibited adaptive immune functions, some of which could be restored by neutralisation of IL-10. Mf lysate-modulated macrophages had reduced phagocytic capacity, while macrophages differentiated in the presence of Mf lysate displayed significantly inhibited innate responses to LPS stimulation. This suggests that microfilariae modulate the innate response by acting on macrophage differentiation and macrophages themselves, and modulate the adaptive CD4^+^ T cell response by acting on monocytes. The phenotypic pattern observed by modulated monocytes is recapitulated *in vivo* in active infection. This highlights a previously unclear mechanism of immune modulation by the parasite.

## Introduction

Lymphatic filariasis is an immune-mediated spectral disease that manifests in two main clinical outcomes: chronic pathology or asymptomatic infection. These outcomes depend on a multitude of factors, including parasite-induced immunoregulation and host genetic background (reviewed elsewhere [1]).

An overt manifestation is the hyperresponsive phenotype that develops in patients with chronic lymphatic pathology (abbreviated as CP). These individuals have increased antigen-specific immunoglobulin (Ig)E and low IgG4 [2], [3], strong T helper (Th)1 and Th17 proinflammatory responses and a greatly diminished T regulatory (Treg) compartment [2], resulting in immunopathological changes in the host. CP patients carry adult worms in the lymphatics but are generally amicrofilaremic, as a strong immune response kills the microfilarial stage. Parasite death leads to the release of antigenic material that triggers inflammation and causes destruction of lymphatic vessels and inflammation [4]. In *Wuchereria bancrofti*, *Brugia malayi* and *B. timori* infections, this can result in the development of elephantiasis or hydrocoele, whereby the lymphatic tissue becomes dilated and hypertrophic.

The second clinical manifestation is a hyporesponsive phenotype characterised by asymptomatic infection (abbreviated as AS), which tolerates the presence of fecund adult worms due to strong parasite-induced immunosuppression and immunomodulation [5]. Importantly, adult worms are tolerated and circulating blood microfilariae are carried by these patients, ensuring transmission. This group has increased numbers of regulatory cells, high interleukin (IL)-10 and elevated levels of antigen-specific IgG4 leading to a modified Th2 response that protects the host and permits parasite survival [5]. Thus, parasite-induced immunomodulation allows persistent infection and continuous transmission while simultaneously enabling the host to tolerate infection by diminishing clinical symptoms. The proportion of individuals in a filaria-endemic area who do not develop one of these two clinical manifestations remain infection- and disease-free and are putatively immune; these individuals are known as endemic normals (abbreviated as EN) [1].

Filaria have been shown to act on host dendritic cells, monocytes, macrophages, T cells and B cells to cause immunomodulation, typically inducing Th2 type and regulatory responses [6]. Immunomodulation occurs through production of specific parasite-derived products that target mammalian host immune cells and signalling pathways. This is strictly dependent on live parasites as shown by the recovery of cellular responsiveness in patients treated with microfilaricidal chemotherapy, specifically diethylcarbamazine (DEC) [7]. While some adults are killed by DEC treatment, the main target is the microfilarial stage, suggesting a prominent role for microfilariae in modulating immune responses [8].

Monocytes and macrophages have long been described to develop a particular phenotype in filarial infection that may contribute to the dysfunction of adaptive immune responses. The spectrum of phenotypes and functions that monocytes and macrophages can develop is vast, and ranges from classical activation, through to alternative activation, wound healing, parasite resistance and immune regulation (reviewed elsewhere [9]). Thus classical activation is characterised by production of proinflammatory molecules and typically develops in response to stimulation with lipopolysaccharide (LPS) plus a secondary stimulus such as interferon (IFN)-γ or tumour necrosis factor (TNF)-α. In contrast alternative activation is induced by stimulation with IL-4 and/or IL-13 and induces macrophages that are involved in wound healing and immune regulation [9]. Murine macrophages that develop in response to these cytokines express specific markers, including arginase (arg)-1, resistin-like molecule (RELM)-α, Ym-1, Ym-2, acidic mammalian chitinase (AMCase), and mannose receptor C type (MRC)-1 [9]. This is a phenotype that is reflected in macrophages in filarial infections [10]. In contrast to the murine system, human monocytes and macrophages typically show a diverse phenotype after stimulation with IL-4, defined by expression of the scavenger receptor CD163 and the chemokine ligand CCL18 [11], [12]. MRC-1 (CD206), similar to mice, is also expressed by human macrophages in this setting [12]. In human filarial infections, expression of arg-1, programmed death-ligand (PD-L)1 and PD-L2 on monocytes has also been reported [13], [14].

Macrophages recruited in filarial infection induce specific hyporesponsiveness in T cells [15], [16]. Macrophages recruited during *B. malayi* infection drive CD4^+^ Th2 responses, deviating the immune system from inducing a proinflammatory Th1 response that could be detrimental to parasite survival [17]. During patent filarial infection monocytes encounter the microfilarial lifecycle stage in the blood, before migrating out to the tissues. This initial contact with the parasite is particularly interesting as it occurs only in AS patients where the adult worms are tolerated in the lymphatics and produce viable microfilariae [18].

We hypothesised that microfilariae in circulation act on human monocytes during patent infection. This primary contact may affect monocyte differentiation, macrophage development and the ensuing innate and adaptive responses and thus may contribute to the development and maintenance of asymptomatic infection. Therefore we aimed to characterise the phenotype of monocytes and macrophages stimulated *in vitro* with *B. malayi* microfilarial (Mf) lysate, and to determine the effect of *B. malayi* Mf lysate-stimulated monocytes and macrophages on defined innate or adaptive functions. We could show that monocytes stimulated with *B. malayi* Mf lysate *in vitro* develop a defined regulatory phenotype, characterised by expression of the immunoregulatory markers IL-10 and PD-L1. Significantly, this regulatory phenotype could be recapitulated in monocytes from *Wuchereria bancrofti* AS patients in contrast to CP patients and EN individuals. Monocytes from non-endemic normal donors stimulated with Mf lysate directly inhibited CD4^+^ T cell proliferation and cytokine production (IFN-γ, IL-13 and IL-10). Importantly, IFN-γ responses could be restored by neutralisation of IL-10 or PD-1. Furthermore, macrophages stimulated with Mf lysate expressed high levels of IL-10 and had suppressed phagocytic abilities. Finally we could show that Mf lysate applied during the differentiation process of macrophages *in vitro* interfered with macrophage abilities to respond to subsequent LPS stimulation in a selective manner.

## Materials and Methods

### Ethics statement

All experiments with material from filaria non-endemic normal donors were approved by the ethical committee of the Charité, Berlin (permit number EA1/104/14). All experiments with material from *W. bancrofti*-exposed donors were approved by the ethical committee of the Blue Peter Public Health and Research Center-LEPRA Society, Hyderabad (permit number 5/2009). Informed written consent was obtained from all participants. All *W. bancrofti* infected donors were treated for lymphatic filariasis by administration of DEC and symptomatic relief after completion of the study. The study was performed according to the Declaration of Helsinki.

### Filarial lysate preparation

Live *B. malayi* microfilariae and adult female worms were a kind donation from the NIAID/NIH Filariasis Research Reagent Resource Center (FR3) in Athens, Georgia. Microfilariae and adult female worms were washed twice in RPMI medium containing 200 U/ml penicillin and 200 µg/ml streptomycin. To collect excretory/secretory (ES) products, microfilariae were cultured for 3–5 days in RPMI containing 1% glucose, 200 U/ml penicillin and 200 µg/ml streptomycin in a 5% CO_2_-incubator at 37°C in 6 well plates whereby media was replaced every 24 h. The resulting ES-containing media was concentrated using Vivacell 70 concentrators (Sartorius Stedim Biotech GmbH, Göttingen, Germany) with a membrane cut-off at 5,000 molecular weight. To prepare microfilarial (Mf) lysate, live microfilariae in suspension were subsequently harvested, washed twice in phosphate buffered saline (PBS) by centrifuging for 10 min at 1500 rpm. Pelleted microfilariae or adult female worms were homogenised directly in a glass homogeniser and ultrasonicated on ice at an intensity of 10% for 3 min. The homogenate was centrifuged at 12,000 rpm and 4°C for 10 min and sterile filtered through a 0.22 µm filter. Protein concentration was determined using the Pierce BCA protein assay kit (Thermo Scientific, Waltham, USA) as per the manufacturer's guidelines. LPS concentration was determined by *Limulus* amoebocyte lysate endotoxin detection kit QCL-1000 (Lonza, Walkersville, USA); the LPS content in *B. malayi* Mf lysate, ES or adult female (Fem) lysate used in all assays was <1 EU/ml in the final concentration.

### Human study populations

*In vitro* experiments using samples from filaria non-endemic normal donors were performed in Germany, using buffy coats purchased from the German Red Cross. Experiments using samples from filaria-exposed donors examined a cohort of 56 individuals from Andhra Pradesh in South India, where lymphatic filariasis caused by *W. bancrofti* is endemic. Night blood smears were performed with 20 µl blood to detect circulating microfilariae, and the TropBio Og4C3 ELISA (TropBio Pty. Ltd, Townsville, Queensland, Australia) was performed using serum to detect circulating filarial antigen (CFA), as per the manufacturer's instructions. Patients with lymphatic filarial pathology (lymphadenitis, lymphoedema, hydrocoele) were examined as part of a clinical protocol approved by the institutional ethical committee of the Blue Peter Public Health and Research Center-LEPRA Society. Based on these results, 28 individuals were classed as endemic normals (EN), 21 had chronic pathology (CP) and 7 had asymptomatic infection (AS) (**Table 1**). Any AS patient found to be positive for CFA was classed as asymptomatic regardless of the night blood smear result; at 20 µl blood per smear, the test has low sensitivity, giving a cut off value of 50 microfilariae per ml.

**Table 1:** Characteristics of the study cohort in Andhra Pradesh, South India.

	Number (M/F)	Median age (range)	Lymphatic pathology	Mf status	CFA status
**Endemic normal**	28 (15/13)	41 (15–63)	no	negative	negative
**Chronic pathology**	21 (4/17)	55 (34–74)	yes	negative	negative
**Asymptomatic**	7 (6/1)	33 (28–66)	no	negative/positive	positive

M, male; F, female; Mf, microfilaria; CFA, circulating filarial antigen.

### Isolation of peripheral blood mononuclear cells

For *in vitro* experiments using samples from non-endemic normal donors, peripheral blood mononuclear cells (PBMCs) were isolated from buffy coats. For experiments using samples from filaria-exposed donors, PBMCs were isolated from 40 ml blood. In both cases PBMCs were isolated by density centrifugation using Lymphocyte Separation Medium (LSM). Blood from buffy coats was initially diluted in phosphate buffered saline (PBS), two parts blood to one part PBS. Briefly, blood was layered onto LSM and centrifuged at 2500 rpm, at room temperature for 25 min with zero brake and zero accelerator. The interphase was collected and washed in PBS plus 0.2% bovine serum albumin (BSA). Cells were centrifuged at 1500 rpm, 4°C for 10 min, washed in PBS plus 0.2% BSA and centrifuged at 1000 rpm, 4°C for 10 min to remove platelets. Lysis of erythrocytes was performed to remove remaining erythrocytes if necessary. For this, 5 ml of ammonium-chloride-potassium lysis buffer (0.01 M KHCO_3_, 0.155 M NH_4_Cl, 0.1 mM EDTA, pH 7.5) was added to cells for 5 min at room temperature, after which cells were washed in PBS plus 0.2% BSA and centrifuged at 1500 rpm for 10 min.

### Isolation of CD14^+^ monocytes and differentiation to macrophages

To positively select for CD14^+^ monocytes, anti-CD14 beads (Miltenyi, Biotec, Bergisch-Gladbach, Germany) were added for 20 min to PBMCs. A volume of 200 µl beads was used on buffy coats, while 50 µl was used on 40 ml whole blood. In both cases, cells were washed in PBS plus 0.2% BSA, 2 mM EDTA and filtered before separation using a 70 µm filter (Partek, St. Louis, USA). Cells from non-endemic normal donors were separated by an autoMACS classic, using the program ‘possel’ (Miltenyi Biotec, Bergisch-Gladbach, Germany). Cells from filaria-exposed donors were separated using MACS MS columns as per the manufacturer's instructions (Miltenyi Biotec, Bergisch-Gladbach, Germany). After isolation, cells were washed once in PBS plus 0.2% BSA, 2 mM EDTA, centrifuged at 1500 rpm and 4°C for 5 min and transferred into complete RPMI medium (RPMI 1640, 5% AB human serum, 100 U/ml penicillin, 100 mg/ml streptomycin, 1 mM L-glutamine, 1 mM MEM non-essential amino acids, 1 mM sodium pyruvate) for use in subsequent experiments. Macrophages were generated *in vitro* by culturing CD14^+^ monocytes in complete RPMI plus 10 ng/ml M-CSF in 6-well cell culture plates at a cell concentration of 0.33×10^6^/ml and a density of 0.1×10^6^/cm^2^ for 6 days, at 37°C and 5% CO_2_. For macrophages differentiated in the presence of Mf lysate, 20 µg/ml *B. malayi* Mf lysate was added to the supernatant at the beginning of culture.

### *In vitro* stimulation of monocytes and macrophages

Monocytes from filaria-exposed donors were used directly for *ex vivo* RT-PCR analysis or seeded at a concentration of 0.2×10^6^ per well into 96-well flat bottom cell culture plates and stimulated for 24 h *in vitro* with 200 µl stimulus. Monocytes from non-endemic normal donors were seeded at a concentration of 2×10^6^ per well into 24-well cell culture plates and stimulated for 4 h or 24 h *in vitro* with 1 ml stimulus. *In vitro* generated macrophages were washed on day 6 with PBS, and the culture supernatant was replaced with 1 ml stimulus for 24 h. Cells were stimulated with 20 ng/ml IL-4 (Peprotech, Rocky Hill, USA), a combination of 20 ng/ml IFN-γ (Peprotech, Rocky Hill, USA) plus 100 ng/ml LPS (Invivogen, California, USA) or 20 µg/ml *B. malayi* Mf lysate at 37°C and 5% CO_2_. Unstimulated controls were included in all experiments. The supernatant was collected and stored at −20°C for further analysis. Cells were lysed in RNA lysis buffer (Analytik Jena, Jena, Germany) for RT-PCR analysis.

### Cytokine analysis

IL-6, IL-8, IL-10, IL-13, IFN-γ and TNF-α protein were measured using a commercial enzyme-linked immunosorbent assay (ELISA) kit from eBioscience (San Diego, USA). IL-12p40 was measured using an ELISA kit from BioLegend (San Diego, USA). All samples were measured in duplicates. Absorbance was read at 450 nm with background wavelength subtracted at 570 nm using the Synergy HT plate reader from BioTek (Winooski, USA).

Active transforming growth factor (TGF)-β was measured using a TGF-β reporter cell line. Briefly, MFB-F11 cells were incubated for 24 h with cell culture supernatant. The resulting supernatant was assessed using the SEAP Reporter Gene Assay (Roche, Branford, USA) according to the manufacturer's instructions.

### RNA extraction and real-time PCR

RNA was isolated from cells using a commercial kit, following the manufacturer's instructions (innuPREP RNA mini-kit, Analytik Jena, Jena, Germany). The concentration of extracted RNA was determined using the NanoDrop 1000. RNA was reverse-transcribed to cDNA using a high capacity RNA-to-cDNA kit (Life Technologies, Darmstadt, Germany). cDNA was set to a concentration of 3–10 ng/µl, depending on the experiment. Real-time PCR was performed with FastStart Universal SYBR Green Master Mix (Roche Applied Science, Indianapolis, USA) using the ABI 7300 Real-Time PCR (Life Technologies, Darmstadt, Germany). Relative changes in gene expression were calculated with ABI 7300 SDS Software (Life Technologies, Darmstadt, Germany). For monocytes and macrophages from non-endemic normal donors, expression levels of transcripts were normalized to the Ct values of the endogenous housekeeping gene by using the 2^−ΔΔCt^ method [19]. Relative expression of genes in stimulated samples was compared to unstimulated controls (which are set at 1). For monocytes from filarial-exposed donors we chose not to normalise data to the reference group (EN), as there was a large variation in Ct values within the heterogeneous EN group. Thus expression levels of transcripts were normalized to the Ct values of the endogenous housekeeping gene by using the method 1/2^ΔCt^ where ΔCt represents the difference between the target gene and the housekeeping gene. Baseline expression of samples from AS or CP was compared to that of EN. In all experiments ß2-microglobulin was used as a housekeeping gene. The gene-specific primer sequences and accession numbers are shown in **Table S1**.

### Flow cytometry analysis of monocytes

Monocytes and macrophages were analysed for surface expression of HLA-DR, CD80, CD86, PD-L1, PD-L2, CD163, CD206, indoleamine 2,3-dioxygenase (IDO) or CD11b. Cells were treated with FcR Blocking Reagent (Miltenyi Biotec, Bergisch-Gladbach, Germany) and stained with Fixable Viability Dye eFluor 780 (eBioscience, San Diego, USA), and with one or combinations of the following: anti-CD80-PE (clone 2D10.4), anti-CD86-PE (clone IT2.2), anti-CD274-PE (clone MIH1), anti-CD273-PE (clone MIH18), anti-CD163-PE (clone GHI/61), anti-IDO-PE (clone eyedio), anti-CD11b-FITC (clone M1/70) (all from eBioscience, San Diego, USA), anti-CD206-APC (clone 15.2, BioLegend, San Diego, USA) or HLA-DR-APC (clone G46-6, BD, Franklin Lakes, USA). Cells were acquired using the FACSCanto II (BD, Franklin Lakes, USA) and analysed using FlowJo, version 8.8.7 (Tree Star, Ashland USA).

### Isolation of CD4^+^ T cells and CFSE labelling

PBMCs were labelled using the CD4**^+^** T cell Isolation Kit II (Miltenyi Biotec, Bergisch-Gladbach, USA) according to the manufacturer's instructions and sorted on an autoMACS classic (Miltenyi Biotec, Bergisch-Gladbach, USA) using the program ‘deplete’. Untouched CD4^+^ T cells were stained with CFSE and rested in complete RPMI for 24 h at 37°C and 5% CO_2_.

### Monocyte: CD4^+^ T cell coculture

Monocytes were left unstimulated or stimulated with 20 µg/ml *B. malayi* Mf lysate or 20 µg/ml *B. malayi* microfilarial ES for 24 h in a 5% CO_2_-incubator at 37°C in 6-well plates. Monocytes were washed and 1×10^5^ monocytes were cocultured with 5×10^5^ CFSE-labelled CD4**^+^** T cells in 96-well flat bottom plates in the presence of 2 µg/ml soluble anti-CD3 (OKT3, eBioscience, San Diego, USA). After 3–5 days, the supernatant was removed for cytokine analysis and cells were stained with Fixable Viability Dye eFluor 780 (eBioscience, San Diego, USA) and anti-CD4-PE-Cy5 (clone RPA-T4, BioLegend, San Diego, USA). Fixed cells were acquired using the FACSCanto II (BD, Franklin Lakes, USA) and analysed using FlowJo, version 8.8.7 (Tree Star, Ashland USA).

### Monocyte: CD4^+^ T cell neutralisation assay

To determine a role for IL-10 signalling or PD-1-PD-L1 interactions in the suppression of CD4^+^ T cell effector functions induced by Mf lysate, monocytes were left unstimulated or stimulated with 20 µg/ml *B. malayi* Mf lysate for 24 h in a 5% CO_2_-incubator at 37°C in 6-well plates. Monocytes were washed and 1×10^5^ monocytes were cocultured with 5×10^5^ CFSE-labelled CD4**^+^** T cells in 96-well plates in the presence of 2 µg/ml soluble anti-CD3 (OKT3, eBioscience, San Diego, USA) plus neutralising anti-IL-10 antibodies or anti-PD-1 antibodies (both at 10 µg/ml, from eBioscience San Diego, USA). After 3–5 days, the supernatant was removed for cytokine analysis and cells were stained with Fixable Viability Dye eFluor 780 (eBioscience, San Diego, USA) and anti-CD4-PE-Cy5 (clone RPA-T4, BioLegend, San Diego, USA). Fixed cells were acquired using the FACSCanto II (BD, Franklin Lakes, USA) and analysed using FlowJo, version 8.8.7 (Tree Star, Ashland USA).

### LPS stimulation of Mf lysate-differentiated macrophages

To determine the ability of Mf lysate-differentiated macrophages to respond to LPS stimulation, Mf lysate-differentiated macrophages were washed on day 6 of culture and 2×10^5^ cells were stimulated for 24 h with 100 ng/ml LPS (Invivogen, California, USA). The following day supernatants were collected for cytokine analysis by ELISA.

### Phagocytosis assay

Macrophages were generated *in vitro* as above, and used after 6 days incubation. To harvest macrophages, the cells were washed three times in PBS, then 1 ml PBS containing 5 mM EDTA was added. The cells were kept at 4°C for 10 min then scraped off the well using a cell scraper. Macrophages were seeded into a 96-well flat bottom plate (0.2×10^6^ cells per well) in complete RPMI, and stimulated using 20 µg/ml *B. malayi* Mf lysate for 24 h. After 24 h macrophages were washed in PBS three times, after which the prepared fluorescent pHrodo BioParticles suspension was added. pHrodo BioParticles (Life Technologies, Darmstadt, Germany) were prepared beforehand by suspending 2 mg BioParticles in 2 ml of uptake buffer (140 mM NaCl, 2.5 mM KCl, 1.8 mM CaCl_2_, 1.0 mM MgCl_2_, 20 mM HEPES, pH 7.4). The solution was briefly vortexed and then sonicated for 5 min to ensure homogenous dispersal of the particles. 100 µl of the prepared suspension was then added to the cells. Unstimulated macrophages were regarded as a positive control. Cytochalasin D (20 µM) was added as a negative control to unstimulated cells. Cells were incubated with the fluorescent particles for 3 h at 37°C (no CO_2_). Finally the cells were washed three times in PBS and 200 µl of 0.5% formalin was added. Fluorescence was read at 550 nm excitation and 600 nm emission using the Synergy HT plate reader from BioTek (Winooski, USA). The net phagocytosis was calculated as per the manufacturer's instructions, by subtracting the average fluorescence intensity of the negative control from the positive control and all experimental wells. The phagocytosis response to the experimental effector (% phagocytosis) could then be calculated as a percentage of the net positive control phagocytosis (% phagocytosis = net phagocytosis×100/net phagocytosis of positive control.

### Statistical analysis

All statistical analyses were performed using GraphPad Prism version 6.0d (GraphPad Software, Inc., California, USA). In experiments where two paired groups were analysed, Wilcoxon signed-rank test was used to compare a condition to its unstimulated control. In experiments where more than two paired groups were analysed, Friedman's ANOVA was used to determine whether a statistically significant difference existed between any of the conditions and the unstimulated control (significance level p<0.05). In case of significance, the main analysis was followed up with a Wilcoxon signed-rank test between a condition and the unstimulated control, whereby a Bonferroni correction was applied. The Kruskal-Wallis test with Dunn's multiple comparisons post-test was used to determine statistical significance between multiple unpaired groups.

## Results

### Monocytes stimulated *in vitro* with *B. malayi* Mf lysate develop a specific activation phenotype

**Figure 1:**
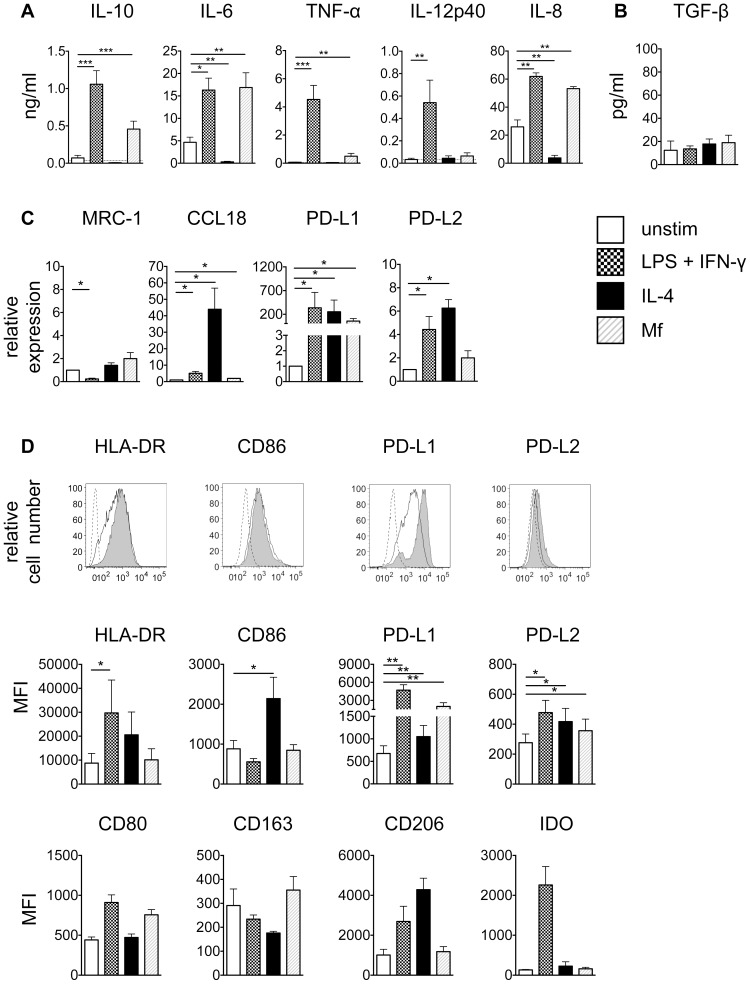
Monocytes stimulated *in vitro* with *B. malayi* Mf lysate develop a specific activation phenotype. Monocytes were left unstimulated or stimulated for 24 h with 100 ng/ml LPS+20 ng/ml IFN-γ, 20 ng/ml IL-4 or 20 µg/ml *B. malayi* Mf lysate. A) Cytokine production was measured using ELISA (pooled data from 4–8 experiments; n = 12–24). Horizontal dashed line indicates the limit of detection of the assay. B) Active TGF-β was measured using a TGF-β reporter assay (pooled data from 2 experiments; n = 6). C) mRNA expression was determined using RT-PCR (pooled data from 3 experiments; n = 8). D) Surface expression of defined markers was measured by flow cytometry. Upper panel, dashed line: FMO control; black line: unstimulated; solid grey: stimulated with Mf lysate. Middle panel, for HLA-DR, CD86, PD-L1 and PD-L2 pooled data from 4 experiments (n = 10) are represented as the mean fluorescence intensity (MFI). Lower panel, for CD80, CD163, CD206 and IDO pooled data from 2 experiments (n = 4–6) are represented as MFI. All data are represented as mean ± SEM. P values were calculated using the Wilcoxon signed-rank test. * p<0.0167, ** p<0.0033, *** p<0.0003.

To understand if *B. malayi* microfilariae act on monocytes to induce immune modulation, monocytes isolated from buffy coats from filaria non-endemic normal donors were stimulated for 24 h *in vitro* with *B. malayi* Mf lysate, LPS plus IFN-γ or IL-4 (**Fig. 1**). Monocytes stimulated with *B. malayi* Mf lysate produced significant and high levels of IL-10, IL-6, TNF-α and IL-8 while IL-12p40 was not induced (**Fig. 1A**). IL-27 was not detected (data not shown). Control stimulation with LPS plus IFN-γ resulted in significantly elevated levels of IL-10, IL-6, TNF-α, IL-8 as well as IL-12p40, which was in stark contrast to Mf lysate-stimulated monocytes. On the other hand, control stimulation with IL-4 significantly inhibited protein production of IL-6 and IL-8 compared to unstimulated controls, while levels of IL-10, TNF-α and IL-12p40 were not significantly altered from unstimulated controls (**Fig. 1A**). Using a TGF-β reporter cell line, the levels of active TGF-β were measured (**Fig. 1B**). Stimulation of monocytes with *B. malayi* Mf lysate, LPS plus IFN-γ or IL-4 did not alter TGF-β production compared to unstimulated controls.

To assess the expression in monocytes of markers associated with an alternative/regulatory phenotype, mRNA expression of *MRC-1* (NM_002438), *CCL18* (NM_002988), *PD-L1* (NM_014143) and *PD-L2* (NM_025239) was analysed after 24 h stimulation (**Fig. 1C**). *B. malayi* Mf lysate induced significantly higher levels of *CCL18* and *PD-L1* compared to unstimulated controls, while the other markers were not altered. Monocytes stimulated with LPS plus IFN-γ or IL-4 upregulated expression of *CCL18*, *PD-L1* and *PD-L2* compared to unstimulated controls. *MRC-1* was significantly inhibited by LPS plus IFN-γ compared to unstimulated controls.

To determine whether the high mRNA level of *PD-L1* was reflected on a protein level, surface expression of PD-L1 was measured by flow cytometry. Expression of the activation markers HLA-DR, CD80 and CD86, the human monocyte alternatively activated markers PD-L2, CD163 and CD206 and the classical activation marker IDO was measured in parallel (**Fig. 1D**). In agreement with the PCR data, PD-L1 was significantly upregulated in monocytes in response to *B. malayi* Mf lysate stimulation (**Fig. 1D**). Significant upregulation of PD-L2 was also observed, however HLA-DR, CD80, CD86, CD163, CD206 and IDO were not significantly different from unstimulated controls. LPS plus IFN-γ induced expression of HLA-DR, PD-L1 and PD-L2, while stimulation with IL-4 upregulated CD86, PD-L1 and PD-L2 (**Fig. 1D**). The other markers analysed were not altered after stimulation with LPS plus IFN-γ or IL-4.

It has previously been shown that microfilariae affect the survival of dendritic cells by apoptosis [20], [21]. To determine whether *B. malayi* Mf lysate affected cell viability, monocytes, *in vitro* generated macrophages or macrophages differentiated in the presence of Mf lysate were stained with a dead cell exclusion dye (**Fig. S1**) and viability was assessed by flow cytometry. There was no difference in the percentage of viable cells in *B. malayi* Mf lysate-stimulated versus unstimulated cells.

### Monocytes from *W. bancrofti* asymptomatically infected donors have a regulatory phenotype at baseline

**Figure 2:**
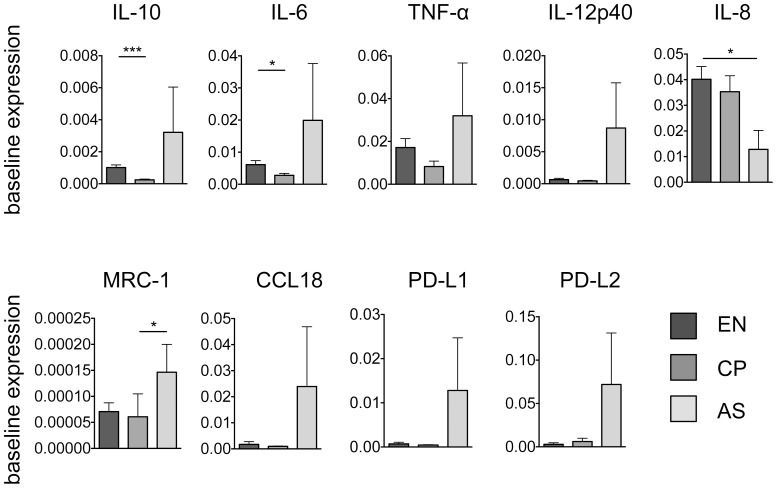
Monocytes from filaria-exposed, asymptomatically infected donors have a regulatory phenotype at baseline. Isolated monocytes from filaria-endemic donors were analysed *ex vivo* for mRNA expression. EN, endemic normal (n = 28); CP, chronic pathology (n = 21); AS, asymptomatic (n = 7). All data are represented as mean ± SEM. P values were calculated using the Kruskal-Wallis test. * p<0.05, *** p<0.001.

Stimulation *in vitro* with *B. malayi* Mf lysate induced a defined regulatory phenotype of monocytes that significantly upregulated the proinflammatory markers IL-6, TNF-α and IL-8 as well as the alternative/regulatory markers IL-10 and PD-L1 in monocytes. Therefore we hypothesised that in filaria-infected individuals, monocytes from asymptomatically infected patients, who exhibit circulating microfilariae in their blood, develop a phenotype similar to that observed in our *in vitro* experiments. Thus, we performed RT-PCR on monocytes isolated from PBMCs from EN, CP and AS donors (**Fig. 2**). There was a trend for monocytes from AS patients to express elevated levels of the alternative/regulatory markers *IL-10* (NM_000572), *MRC-1*, *CCL18*, *PD-L1* and *PD-L2* as well as the proinflammatory markers *IL-6* (NM_000600), *TNF-α* (NM_000594) and *IL-12p40* (NM_002187). *IL-8* (NM_000584) was significantly downregulated in AS patients compared to EN individuals. *MRC-1* was significantly elevated in AS patients compared to CP donors. In contrast, there appeared to be little difference between CP and EN although *IL-10* and *IL-6* were significantly downregulated in CP compared to EN donors. Thus, monocytes isolated from PBMCs from AS patients recapitulate to a great extent the expression profile observed in *B. malayi* Mf lysate-stimulated monocytes from filaria non-endemic donors.

Monocytes from AS patients have been shown to be functionally defective in terms of Toll-like receptor (TLR) expression and function [22], [23]. Thus we wanted to establish the capacity of monocytes from AS patients to respond to *B. malayi* Mf lysate (**Fig. S2**). After 24 h without stimulation in culture, monocytes from EN, CP and AS donors responded equally in terms of protein production of IL-10, IL-6, TNF-α and IL-12p40. After 24 h stimulation with *B. malayi* Mf lysate, monocytes from all three groups responded by producing equal amounts of cytokines. Hence, there was no inherent defect in the ability of monocytes from EN, CP and AS donors to produce cytokines in response to *B. malayi* Mf lysate.

### *B. malayi* Mf lysate-stimulated monocytes impair CD4^+^ T cell proliferation and cytokine production

**Figure 3:**
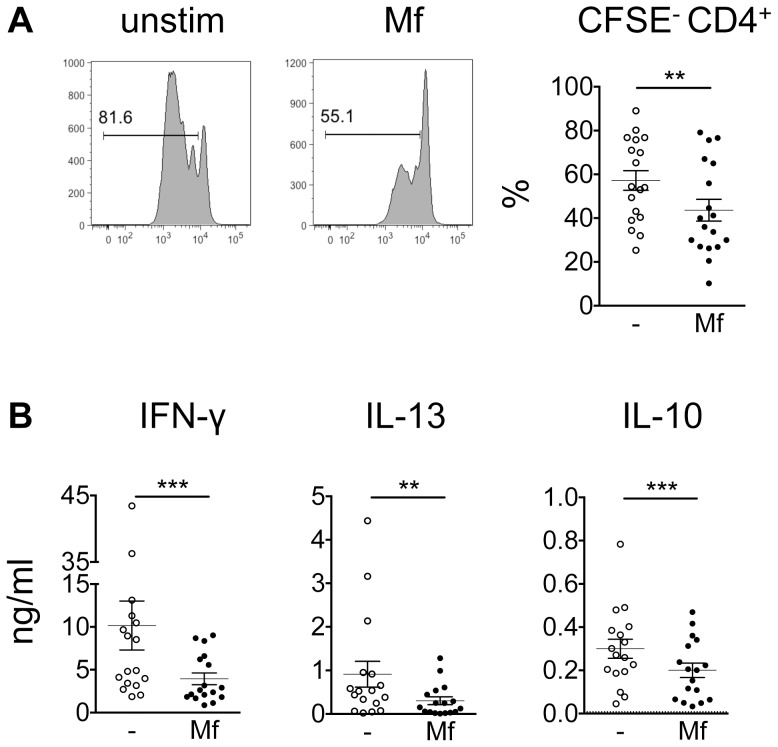
*B. malayi* Mf lysate-stimulated monocytes impair CD4^+^ T cell proliferation and cytokine production. 5×10^5^ CFSE-labelled CD4^+^ T cells were incubated with 1×10^5^ monocytes left unstimulated (open circles) or stimulated for 24 h with 20 µg/ml Mf lysate (closed circles) for 3 to 5 days. A) Flow cytometric analysis of CD4^+^ T cells. Plots show dilution of CFSE over 3–5 day period of assay in T cells coincubated with unstimulated monocytes (left plot) or Mf lysate-stimulated monocytes (right plot). Graph shows the percentage of CD4^+^ T cells that divided. B) Cytokine expression was measured in the culture supernatant by ELISA. Horizontal dashed line indicates the limit of detection of the assay (pooled data from 4–6 experiments; n = 11–18). All data are represented as mean ± SEM. P values were calculated using the Wilcoxon signed-rank test. ** p<0.01, *** p<0.001.

To determine whether the activation phenotype seen in monocytes stimulated with *B. malayi* Mf lysate may account for the hyporesponsiveness of CD4^+^ T cells observed in *ex vivo* studies with PBMCs from AS patients [18], [24]–[27] a coculture assay with autologous CD4^+^ T cells was employed. To this end, autologous CFSE-labelled CD4^+^ T cells were polyclonally stimulated and incubated with *B. malayi* Mf lysate-stimulated monocytes (**Fig. 3**). T cell proliferation was significantly inhibited after coculture with *B. malayi* Mf lysate-stimulated monocytes (**Fig. 3A**). The production of IFN-γ, IL-13 and IL-10 was significantly inhibited in the supernatant of cocultures with *B. malayi* Mf lysate-stimulated monocytes compared to cocultures with unstimulated monocytes (**Fig. 3B**). Thus, Mf lysate-modulated monocytes showed a significantly impaired ability to stimulate CD4^+^ T cell functions.

In order to understand if monocytes treated with microfilaria-derived ES products could alter T cell responses we repeated the coculture but stimulated monocytes with 20 µg/ml *B. malayi* microfilarial ES. Microfilarial ES-stimulated monocytes did not suppress CD4^+^ T cell effector functions (**Fig. S3**).

### Neutralisation of IL-10 and PD-1 restores CD4^+^ T cell IFN-γ production

**Figure 4:**
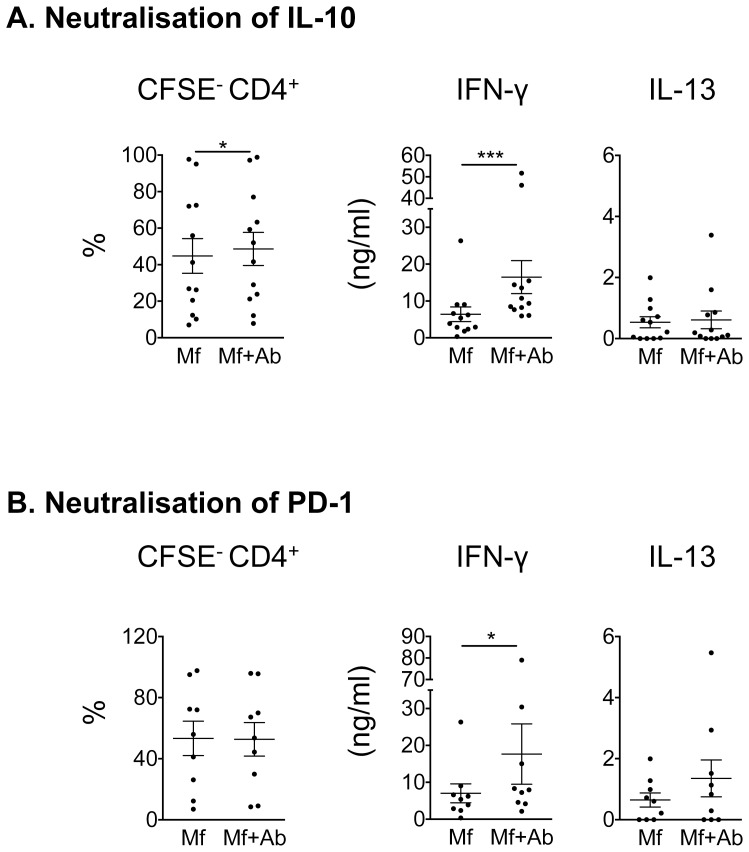
Neutralisation of IL-10 or PD-1 restores CD4^+^ T cell IFN-γ production. 5×10^5^ CFSE-labelled CD4^+^ T cells were incubated for 5 days with 1×10^5^ monocytes stimulated for 24 h with 20 µg/ml Mf lysate +/− 10 µg/ml neutralizing antibodies (Ab) for A) IL-10 (pooled data from 4 experiments; n = 12) or B) PD-1 (pooled data from 3 experiments; n = 9). Proliferation (measured as CFSE dilution) of CD4^+^ T cells was measured by flow cytometry. Cytokine production was measured in the culture supernatant by ELISA. Horizontal dashed line indicates the limit of detection of the assay. All data are represented as mean ± SEM. P values were calculated using the Wilcoxon signed-rank test. * p<0.05, *** p<0.001.

As IL-10 and PD-L1 were significantly upregulated in *B. malayi* Mf lysate-stimulated monocytes, we hypothesised that one of these molecules could be involved in inhibiting CD4^+^ T cell functions as observed in **Fig. 3**. Thus we repeated the coculture experiment and included neutralising anti-IL-10 antibodies to block IL-10 signalling or anti-PD-1 antibodies to block PD-1-PD-L1 interactions (**Fig. 4**). Experiments using unstimulated monocytes in the coculture were performed as a control (**Fig. S4**). While proliferation of T cells was elevated after neutralisation of IL-10 to a statistically significant level (p = 0.016, **Fig. 4A**), the biological difference in restoration was minimal (44.78% of T cells proliferated in response to Mf lysate-stimulated monocytes compared with 48.59% of T cells proliferating after IL-10 was neutralised). Proliferation of T cells was not changed after neutralisation of PD-1 (**Fig. 4B**). IFN-γ production was restored in response to neutralisation of IL-10 (**Fig. 4A**) and PD-1 (**Fig. 4B**), while IL-13 responses were not restored.

To determine whether monocytes were actively expressing IL-10 upon the time of coculture, *B. malayi* Mf lysate-stimulated monocytes were assessed for *IL-10* mRNA expression by RT-PCR (**Fig. S5**). After 24 h stimulation with *B. malayi* Mf lysate, there was a trend for monocytes to express IL-10 mRNA, although this did not reach statistical significance.

### Macrophages stimulated *in vitro* with *B. malayi* Mf lysate develop a specific activation phenotype

**Figure 5:**
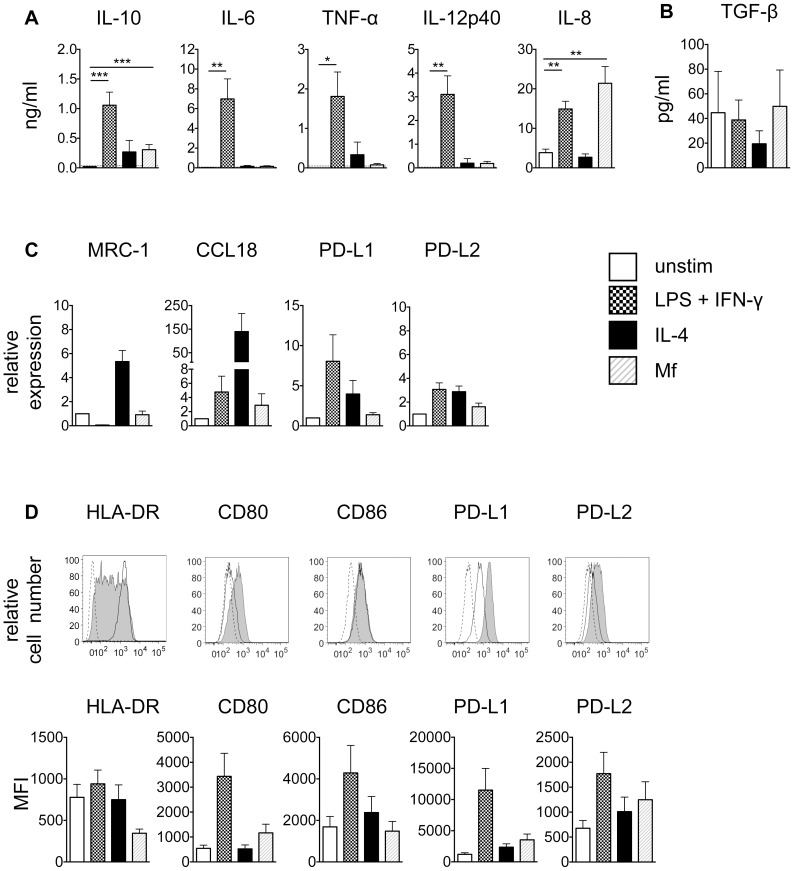
Macrophages stimulated *in vitro* with *B. malayi* Mf lysate develop a specific activation phenotype. Macrophages were left unstimulated or stimulated for 24 h with 100 ng/ml LPS+20 ng/ml IFN-γ, 20 ng/ml IL-4 or 20 µg/ml Mf lysate. A) Cytokine production was measured using ELISA (pooled data from 4–6 experiments; n = 8–18). Horizontal dashed line indicates the limit of detection of the assay. B) Active TGF-β was measured using a TGF-β reporter assay (pooled data from 2 experiments; n = 6). C) mRNA expression was determined using RT-PCR (pooled data from 2 experiments; n = 6). D) Surface expression of HLA-DR, CD80, CD86, PD-L1 and PD-L2 was measured by flow cytometry. Upper panel, dashed line: FMO control; black line: unstimulated; solid grey: stimulated with Mf lysate. Lower panel, pooled data from 2 experiments (n = 6) are represented the mean fluorescence intensity (MFI). All data are represented as mean ± SEM. P values were calculated using the Wilcoxon signed-rank test. * p<0.0167, ** p<0.0033, *** p<0.0003.

To understand if *B. malayi* microfilariae act on macrophages to induce immune modulation, monocytes isolated from buffy coats from filaria non-endemic normal donors were differentiated to macrophages *in vitro* and stimulated for 24 h *in vitro* with *B. malayi* Mf lysate, LPS plus IFN-γ or IL-4 (**Fig. 5**). Similar to monocytes (see **Fig. 1A**), stimulation with *B. malayi* Mf lysate led macrophages to produce significantly higher levels of IL-10 and IL-8 compared to unstimulated controls, while IL-6, TNF-α and IL-12p40 were not induced (**Fig. 5A**). IL-27 was not detected (data not shown). This is in clear contrast to stimulation with LPS plus IFN-γ, which led to significant and high expression of IL-10, IL-6, TNF-α, IL-12p40 and IL-8. IL-4 stimulation did not induce significant production of any cytokines measured **(** **Fig. 5A**). Using a TGF-β reporter cell line, the levels of active TGF-β were measured (**Fig. 5B**). Stimulation of macrophages with *B. malayi* Mf lysate, LPS plus IFN-γ or IL-4 did not alter production of TGF-β compared to unstimulated controls. On mRNA level, macrophages stimulated with *B. malayi* Mf lysate did not alter expression of *MRC-1, CCL18*, *PD-L1* or *PD-L2* compared to unstimulated controls (**Fig. 5C**). There were no significant changes in expression of these markers after stimulation of macrophages with LPS plus IFN-γ or IL-4 compared to unstimulated controls (**Fig. 5C**).

In terms of cell surface markers, stimulation with *B. malayi* Mf lysate did not alter expression of HLA-DR, CD80, CD86, PD-L1 or PD-L2 in macrophages compared to unstimulated controls (**Fig. 5D**). A similar result was observed in macrophages stimulated with LPS plus IFN-γ or IL-4 (**Fig. 5D**).

### *B. malayi* Mf lysate interferes with the differentiation of macrophages *in vitro*

**Figure 6:**
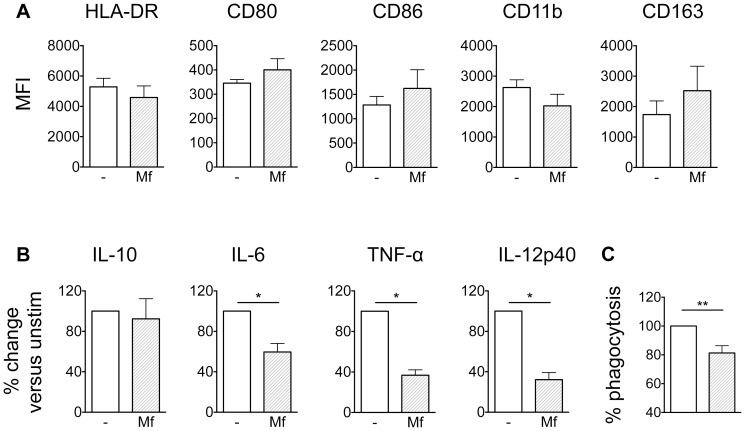
*B. malayi* Mf lysate alters specific innate immune responses of macrophages. Monocytes were differentiated to macrophages with M-CSF in the absence or presence of 20 µg/ml Mf lysate. A) After 6 days macrophages were analysed for surface expression of macrophage maturation markers by flow cytometry (pooled data from 2 experiments; n = 6). B) After 6 days the cell culture supernatant was removed and replaced with fresh medium plus 100 ng/ml LPS for 24 h, to assess the capacity of Mf lysate-differentiated macrophages to produce cytokines, as measured by ELISA (pooled data from 2 experiments; n = 6). C) Monocytes were differentiated to macrophages with M-CSF for 6 days, after which cells were left unstimulated or stimulated for a further 24 h with Mf lysate. The phagocytic capacity of macrophages was determined by measuring the phagocytosis of fluorescently labelled bioparticles (pooled data from 5 experiments; n = 14). All data are represented as mean ± SEM. P values were calculated using the Wilcoxon signed-rank test. * p<0.05, ** p<0.01.

It is feasible that microfilariae interfere with the differentiation of monocytes to macrophages *in vivo* during patent infection, due to their shared anatomical locations. Thus we determined the phenotype and functions of macrophages generated *in vitro* from CD14^+^ monocytes in the presence of 20 µg/ml *B. malayi* Mf lysate (**Fig. 6**). Mf lysate-differentiated macrophages did not alter expression of the maturation markers HLA-DR, CD80 and CD86 or the macrophage markers CD11b and CD163 compared to macrophages generated in the absence of Mf lysate (**Fig 6A**). However when macrophages generated in the presence of Mf lysate were washed and stimulated with 100 ng/ml LPS, there was a significant and selective inhibition of IL-6, TNF-α and IL-12p40 but not IL-10, when compared to macrophages generated in the absence of Mf lysate (**Fig. 6B**).

### *B. malayi* Mf lysate-stimulated macrophages display impaired phagocytosis

To determine whether phagocytosis of macrophages was modulated by stimulation with *B. malayi* Mf lysate, *in vitro* generated macrophages were stimulated for 24 h with *B. malayi* Mf lysate. Subsequently the phagocytic capacity was determined by measuring the phagocytosis of fluorescently labelled bioparticles, whereby unstimulated cells were used as a positive control (**Fig. 6C**). *B. malayi* Mf lysate significantly inhibited phagocytosis in macrophages, reducing this function by approximately 20%.

## Discussion

Our data show that *B. malayi* Mf lysate induces a regulatory monocyte phenotype that curtails CD4^+^ T cell effector functions *in vitro*. This monocyte population is characterized by expression of IL-10 and PD-L1 as well as certain proinflammatory markers. This regulatory phenotype is reflected in monocytes from AS patients with active filarial infection, but not in CP patients or EN donors. Importantly, CD4^+^ T cell IFN-γ responses could be recovered after neutralisation of IL-10 or PD-1. Furthermore we could show that macrophages stimulated with Mf lysate expressed high levels of IL-10 and had suppressed phagocytic abilities. Finally, Mf lysate applied during the differentiation process of macrophages *in vitro* interfered with macrophage abilities to respond to subsequent LPS stimulation in a selective manner. A limitation to our results from filaria-exposed donors was the access to only very low numbers of AS donors used for analysis of *ex vivo* monocyte phenotype and for stimulation of filaria-endemic monocytes *in vitro*. Such low numbers of AS patients are in part due to the extensive mass drug treatment effort in South India during the last decade [28], [29].

The observed monocyte phenotype is in accordance with a previous study that described monocytes stimulated with live microfilariae to upregulate PD-L1 and to have an alternatively activated phenotype [14]. Monocytes isolated from filarial lysate-treated PBMCs from humans with asymptomatic filarial infection are characterised by increased expression of arg-1 and IL-10 and decreased levels of nitric oxide synthase (NOS, typically used as a classical activation marker in murine studies) compared to endemic normal controls [13]. Interestingly in our studies inducible NOS (iNOS) could not be detected in *B. malayi* Mf lysate-treated monocytes or macrophages *in vitro* or in monocytes from endemic patients *ex vivo* (data not shown). iNOS historically can be difficult to detect, thus the differences observed here may lie in the techniques used [30]. Furthermore murine markers cannot simply be translated into the human system [31], [32]. As an example, reports indicate that arg-1 may not be a reliable marker for alternatively activated monocytes or macrophages in humans as it is found in other cell types [33]. Human monocytes do not express arg-1 after stimulation with IL-4 and IL-13 (the prototypical inducers of alternative activation), unlike mouse macrophages [31]. Thus we have carefully selected a number of markers that have previously been reported for classical activation, alternative activation, or regulation in human monocytes and macrophages [9], [11], [12], [31], [34]. We have used these as the basis for characterising the cells in the current study.

Our results indicate a phenotype that consists of a spectrum of proinflammatory, alternatively activated and regulatory markers, which is in line with numerous reports stating the mixed Th1/Th2 response that is often seen after exposure to microfilariae [18], [20], [35]–[37]. The mixture of markers observed may be partly influenced by *Wolbachia*, the obligate endosymbiont found in all lifecycle stages of the filarial parasites *W. bancrofti* and *B. malayi*. *Wolbachia* are known to induce inflammatory responses in monocytes in a TLR4-independent manner [38]–[40]. Contrary to this, monocytes treated with lysate from adult female worms do not upregulate any of the cytokines tested. This highlights a microfilaria-derived factor other than *Wolbachia* to be responsible for the effects seen here with Mf lysate-treated monocytes (**Fig. S6**). Importantly in our studies, Mf lysate did not induce production of IL-12p40, in stark contrast to stimulation of monocytes or macrophages with LPS plus IFN-γ. Similarly there was no detection of TNF-α in Mf lysate-stimulated macrophages, which was highly induced by LPS plus IFN-γ. Monocytes showed some production of TNF-α however this was approximately five times less than that observed after stimulation with LPS plus IFN-γ. On the other hand, the proinflammatory markers as well as IL-10 were not detected in either monocytes or macrophages after stimulation with IL-4. Thus Mf lysate produces a clear and distinct response to that induced by the hallmark proinflammatory stimulus, LPS plus IFN-γ or the prototypical alternative activation stimulus, IL-4.

Nevertheless it is unclear to what extent the expression of proinflammatory cytokines may function in the face of high levels of immunoregulatory cytokines such as IL-10. We have previously shown that IL-10 is induced in murine macrophages by a nematode-derived cysteine-proteinase inhibitor isolated from a related filarial species, *Acanthocheilomena viteae* (AvCystatin) [41]. Thus it would be interesting to investigate the role of filarial cystatin in *B. malayi* Mf lysate-induced IL-10 expression, as *B. malayi* contains three different forms of cystatin, with Bm-CPI-2 being more closely related to AvCystatin [42]. Furthermore, AvCystatin was shown to mediate IL-10- and macrophage-dependent immunomodulation in a mouse model of airway hyperreactivity [43]. Thus, future experiments should determine the contribution of *B. malayi* cystatins to human T cell hyporesponsiveness.

Establishing the phenotype and function of *B. malayi* Mf lysate-stimulated monocytes and macrophages from non-endemic normal donors highlights these cells as instruments of microfilarial immune modulation. To elucidate the exact phenotype of monocytes during infection, it was necessary to analyse the cytokine and marker profile of monocytes from endemic individuals *ex vivo*, without prior stimulation in the presence of other immune cells as done in other studies [13]. Therefore we examined monocytes from individuals with *W. bancrofti* asymptomatic infection that had presumably interacted with microfilariae in circulation in the 12 hours prior to isolation. As expected, in the absence of any external stimulation, only monocytes from this group produced the specific phenotype that was previously observed *in vitro*. Nevertheless in response to specific stimulation, we found that monocytes from all three groups responded with a cytokine profile that closely reflected that seen under the same conditions in monocytes from filaria non-endemic normal controls. This observation revealed that monocytes from all filaria-exposed donors principally had the capacity to react to Mf lysate without an inherent defect in one of the patient groups.

Monocytes and macrophages that develop in helminth infections are believed to contribute to wound healing, regulation of Th1 and Th2 inflammation and expulsion of the parasite from the host (reviewed elsewhere [44]). Asymptomatically infected patients are the only filarial-exposed group in which monocytes in the blood come into contact with live microfilariae; thus monocytes may be influenced at this early time point in their differentiation to contribute to immune regulation and therefore the development of asymptomatic infection. Indeed it has been shown that *B. malayi* microfilariae act on monocytes from filaria non-endemic normal donors to reduce transendothelial migration [45]. To this end, we established that monocytes and macrophages from non-endemic normal donors stimulated with *B. malayi* Mf lysate *in vitro* develop a specific phenotype upon activation, and that these cells may influence either the adaptive or innate immune response, respectively.

*B. malayi* Mf lysate-stimulated monocytes could suppress CD4^+^ T cell proliferation as well as IFN-γ and IL-13 cytokine production in an autologous coculture assay. T cells that received a polyclonal stimulus in the presence of *B. malayi* Mf lysate-treated monocytes displayed significantly reduced proliferation compared to T cells stimulated in the presence of control monocytes. Furthermore, their ability to produce effector cytokines was significantly inhibited. This is in line with a previous report that demonstrated that PBMCs from microfilaremic patients produce fewer Th1 and Th2 cytokines when stimulated with live microfilariae compared to PBMCs from endemic normals [24].

PD-L1 and IL-10 were significantly induced by Mf lysate stimulation of monocytes and macrophages and thus represented prime candidates responsible for the suppression of T cell responses. Interestingly, neutralisation of IL-10 or PD-1 led to a recovery of CD4^+^ T cell IFN-γ but not IL-13 production. IL-10 has previously been described to be upregulated in adherent cells from patients with lymphatic filariasis [46] and in monocytes from patients harbouring tissue-dwelling filaria [38]. IL-10 has a well-defined role in filarial infections as an immunoregulatory cytokine that regulates both Th1- and Th2-derived inflammatory, potentially harmful responses [47]. PBMCs from asymptomatically infected patients spontaneously secrete significantly higher levels of IL-10 than PBMCs from patients with chronic pathology [46]. Induction of IL-10 is also associated with high levels of immune regulatory IgG4 in human asymptomatic filarial infection (reviewed elsewhere [4]). Thus the high levels of IL-10 observed in our study that resulted from *B. malayi* Mf lysate stimulation may contribute to T cell suppression in asymptomatic infection.

PD-L1 has been described on monocytes stimulated with live microfilariae *in vitro* [14]. PD-L1 together with its receptor PD-1 has an important role as a negative costimulator in numerous infection settings [48]. The high mRNA and surface expression of PD-L1 on *B. malayi* Mf lysate-stimulated monocytes support the idea that microfilaria-modulated monocytes may contribute to asymptomatic infection through this mechanism. Indeed IFN-γ responses were also restored after neutralization of PD-1, supporting a role for this molecule.

Neutralisation of IL-10 or PD-1 had only a minimal effect on the recovery of proliferation and had no effect on the restoration of IL-13, implying that other inhibitory mechanisms may be involved. Van der Werf *et al.* have previously shown that the PD-1-PD-L2 pathway is responsible for Th2 cell hyporesponsiveness in *L. sigmodontis* infection [49]. Nevertheless this pathway would have been similarly neutralised in our assays through application of the anti-PD-1 antibody, suggesting that other mechanisms play a role in the human T cell impairment observed in our experiments. Other candidates that have been described in murine literature to suppress T cell responses in helminth or other Th2-related diseases through a monocyte/macrophage interaction include arginase [50], [51] and RELM-α [52], [53], however as mentioned above, these may not represent reliable options in the human system. Further studies describe the roles of retinoic acid or TGF-β in directing the development of Treg cells [54]–[56]; whether the CD4^+^ T cells in our system have a regulatory phenotype should be further investigated.

In response to stimulation with *B. malayi* Mf lysate, macrophages upregulated high levels of IL-10, but in contrast to monocytes no significant upregulation of PD-L1 expression was detected. However when *B. malayi* Mf lysate was added during the differentiation process of macrophages there was a significant and selective impairment in the ability of macrophages to produce cytokines in response to LPS stimulation. Interestingly this was not caused by a reduction in cell survival or a change in cell activation markers. Similar results have been published by Semnani *et al.* for monocyte-derived dendritic cells stimulated with microfilarial antigen during the differentiation process [57]. Importantly, our results demonstrated that subsequent stimulation with LPS of macrophages differentiated in the presence of Mf lysate resulted in diminished IL-6, TNF-α and IL-12p40 production but not IL-10. This could be a result of distinct signalling pathways being activated in Mf lysate-differentiated macrophages and should be a subject of further studies. The selective impairment of expression of proinflammatory cytokines hints towards a possible involvement of NF-κB1 p50 homodimers, as shown previously [58].

It has been previously demonstrated that *B. malayi* live microfilariae or live microfilariae in a transwell do not induce phagocytosis in monocytes compared to monocytes exposed to M-CSF for 48 h [14]. The authors of this study argued that microfilariae failed to promote phagocytosis. In contrast we believe that *B. malayi* microfilariae (or, in our case, *B. malayi* Mf lysate) directly inhibit phagocytosis of macrophages as a form of immunomodulation. The location of macrophages in the tissues compared to monocytes in the blood may place macrophages in a more advantageous position to phagocytose. In another study, *W. bancrofti* endemic normal monocytes were incubated with serum from the different groups (endemic normal, patients with lymphatic pathology, microfilaremic patients) [23]. Only monocytes incubated with serum from microfilaremic patients had reduced levels of spreading but not phagocytosis. Thus future studies should determine in detail whether inhibition of phagocytosis actually translates to an increase in microfilarial survival, and which other cells or serum components contribute to microfilarial killing and phagocytosis *in vivo*.

In conclusion this study has elucidated the monocyte phenotype in patients with active filarial infection and the regulatory capacity of this cell. By directly acting on monocytes in the blood, microfilariae may regulate the antigen-specific T cell response. Furthermore microfilariae may interfere with the differentiation process of macrophages, thus altering their ability to respond to unrelated stimuli in the tissues. The extent to which these findings promote parasite survival and transmission is unclear and should be further investigated.

## Supporting Information

Figure S1 ***B. malayi*** **Mf lysate does not affect cell viability.** A) Monocytes were left unstimulated or stimulated for 24 h with LPS+IFN-γ, IL-4 or Mf lysate. B) Monocytes were differentiated to macrophages for 6 days with M-CSF and then were left unstimulated or stimulated for 24 h with LPS+IFN-γ, IL-4 or Mf lysate. C) Monocytes were differentiated to macrophages for 6 days with M-CSF in the presence of LPS+IFN-γ, IL-4 or Mf lysate. A dead cell exclusion dye was used to stain cells that were subsequently acquired by flow cytometry (pooled data from 2 experiments; n = 6). All data are represented as mean ± SEM. P values were calculated using the Wilcoxon signed-rank test. * p<0.0167.
(TIF)

Figure S2 ***B. malayi*** **Mf lysate acts on monocytes regardless of the immunological background of the host.** Monocytes from filaria-endemic donors were stimulated for 24 h with Mf lysate, after which cytokine production in the supernatant was measured by ELISA. EN, endemic normal (n = 14); CP, chronic pathology (n = 20); AS, asymptomatic infection (n = 4). Horizontal dashed line indicates the limit of detection. Data are represented as mean ± SEM. P values were calculated using the Kruskal-Wallis test. ns, not significant.
(TIF)

Figure S3 **Monocytes stimulated with excretory/secretory (ES) products from live** ***B. malayi*** **microfilariae do not impair CD4^+^ T cell proliferation or cytokine production.** 5×10^5^ CFSE-labelled CD4^+^ T cells were incubated with 1×10^5^ monocytes left unstimulated (open circles) or stimulated for 24 h with *B. malayi* microfilarial ES (closed circles) for 3 to 5 days. Proliferation (measured as CFSE dilution) of CD4^+^ T cells was measured by flow cytometry. Cytokine expression was measured in the culture supernatant by ELISA. Horizontal dashed line indicates the limit of detection of the assay (pooled data from 2 experiments; n = 5–6). All data are represented as mean ± SEM. P values were calculated using the Wilcoxon signed-rank test.
(TIF)

Figure S4 **Neutralisation of IL-10 in cocultures with unstimulated monocytes.** 5×10^5^ CFSE-labelled CD4^+^ T cells were incubated for 5 days with 1×10^5^ unstimulated monocytes +/− neutralizing antibodies (Ab) for A) IL-10 (pooled data from 4 experiments; n = 12) or B) PD-1 (pooled data from 3 experiments; n = 9). Proliferation (measured as CFSE dilution) of CD4^+^ T cells was measured by flow cytometry. Cytokine production was measured in the culture supernatant by ELISA. Horizontal dashed line indicates the limit of detection of the assay. All data are represented as mean ± SEM. P values were calculated using the Wilcoxon signed-rank test. ** p<0.01, *** p<0.001.
(TIF)

Figure S5 ***B. malayi*** **Mf lysate-stimulated monocytes express IL-10 mRNA at the time of coculture.** Monocytes were stimulated for 4 h or 24 h with *B. malayi* Mf lysate. mRNA expression was determined using RT-PCR (pooled data from 3 experiments; n = 9). All data are represented as mean ± SEM. P values were calculated using the Wilcoxon signed-rank test. * p<0.05.
(TIF)

Figure S6 **Mf lysate but not Fem lysate induces cytokine production in human monocytes.** Human monocytes were stimulated for 24 h with 20 µg/ml microfilarial (Mf) or female (Fem) lysate and cytokines were assessed in the supernatant (pooled data from 4–8 experiments; n = 12–24). Data are represented as mean ± SEM. P values were calculated using the Wilcoxon signed-rank test. ns, not significant. ** p<0.005.
(TIF)

Table S1 **Primer pair sequences used for real-time PCR.**
(DOCX)
