# Supplementary figures and images for: Brugia malayi Microfilariae Induce a Regulatory Monocyte/Macrophage Phenotype That Suppresses Innate and Adaptive Immune Responses
Source: PLoS Negl Trop Dis. 2014 Oct 2;8(10):e3206. doi: 10.1371/journal.pntd.0003206 (PMC4183501; doi:10.1371/journal.pntd.0003206)

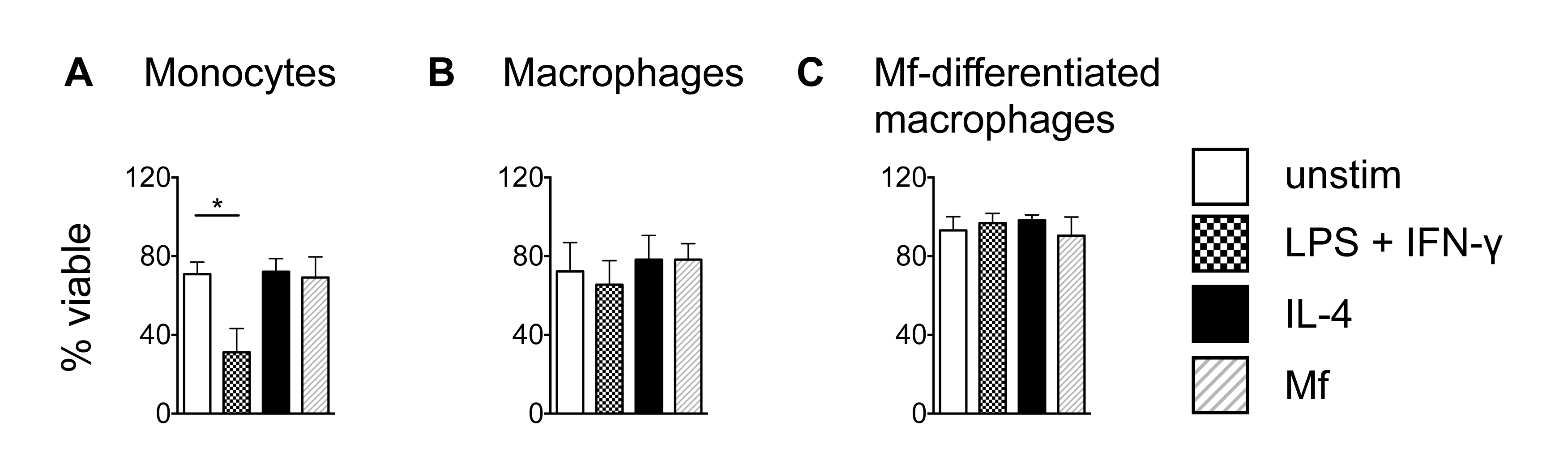

Supplement: Figure S1 — B. malayi Mf lysate does not affect cell viability. A) Monocytes were left unstimulated or stimulated for 24 h with LPS+IFN-γ, IL-4 or Mf lysate. B) Monocytes were differentiated to macrophages for 6 days with M-CSF and then were left unstimulated or stimulated for 24 h with LPS+IFN-γ, IL-4 or Mf lysate. C) Monocytes were differentiated to macrophages for 6 days with M-CSF in the presence of LPS+IFN-γ, IL-4 or Mf lysate. A dead cell exclusion dye was used to stain cells that were subsequently acquired by flow cytometry (pooled data from 2 experiments; n = 6). All data are represented as mean ± SEM. P values were calculated using the Wilcoxon signed-rank test. * p<0.0167. (TIF) [file pntd.0003206.s001.tif]

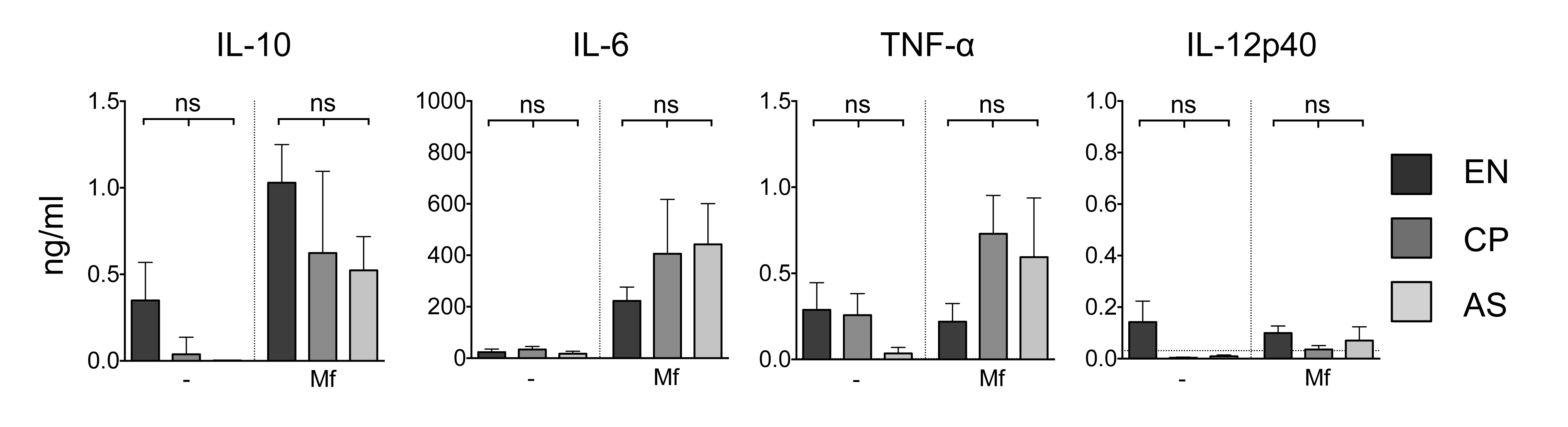

Supplement: Figure S2 — B. malayi Mf lysate acts on monocytes regardless of the immunological background of the host. Monocytes from filaria-endemic donors were stimulated for 24 h with Mf lysate, after which cytokine production in the supernatant was measured by ELISA. EN, endemic normal (n = 14); CP, chronic pathology (n = 20); AS, asymptomatic infection (n = 4). Horizontal dashed line indicates the limit of detection. Data are represented as mean ± SEM. P values were calculated using the Kruskal-Wallis test. ns, not significant. (TIF) [file pntd.0003206.s002.tif]

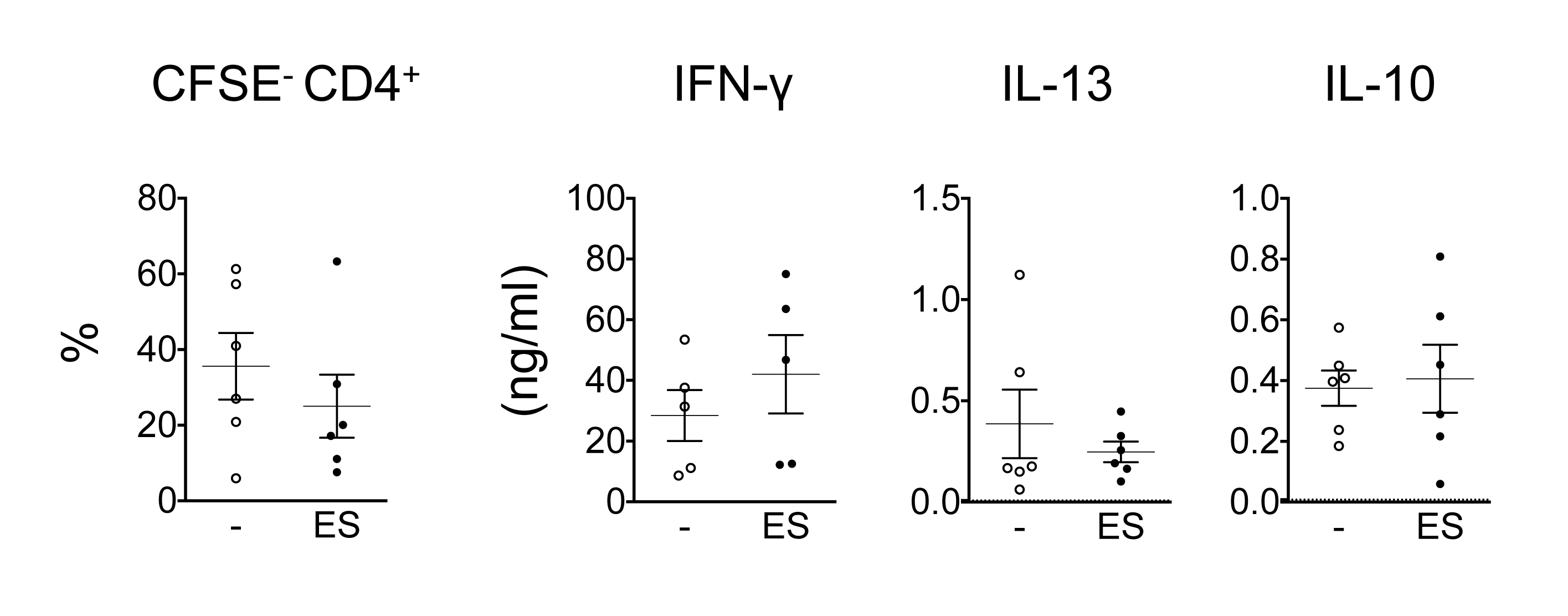

Supplement: Figure S3 — Monocytes stimulated with excretory/secretory (ES) products from live B. malayi microfilariae do not impair CD4+ T cell proliferation or cytokine production. 5×10^5^ CFSE-labelled CD4+ T cells were incubated with 1×10^5^ monocytes left unstimulated (open circles) or stimulated for 24 h with B. malayi microfilarial ES (closed circles) for 3 to 5 days. Proliferation (measured as CFSE dilution) of CD4+ T cells was measured by flow cytometry. Cytokine expression was measured in the culture supernatant by ELISA. Horizontal dashed line indicates the limit of detection of the assay (pooled data from 2 experiments; n = 5–6). All data are represented as mean ± SEM. P values were calculated using the Wilcoxon signed-rank test. (TIF) [file pntd.0003206.s003.tif]

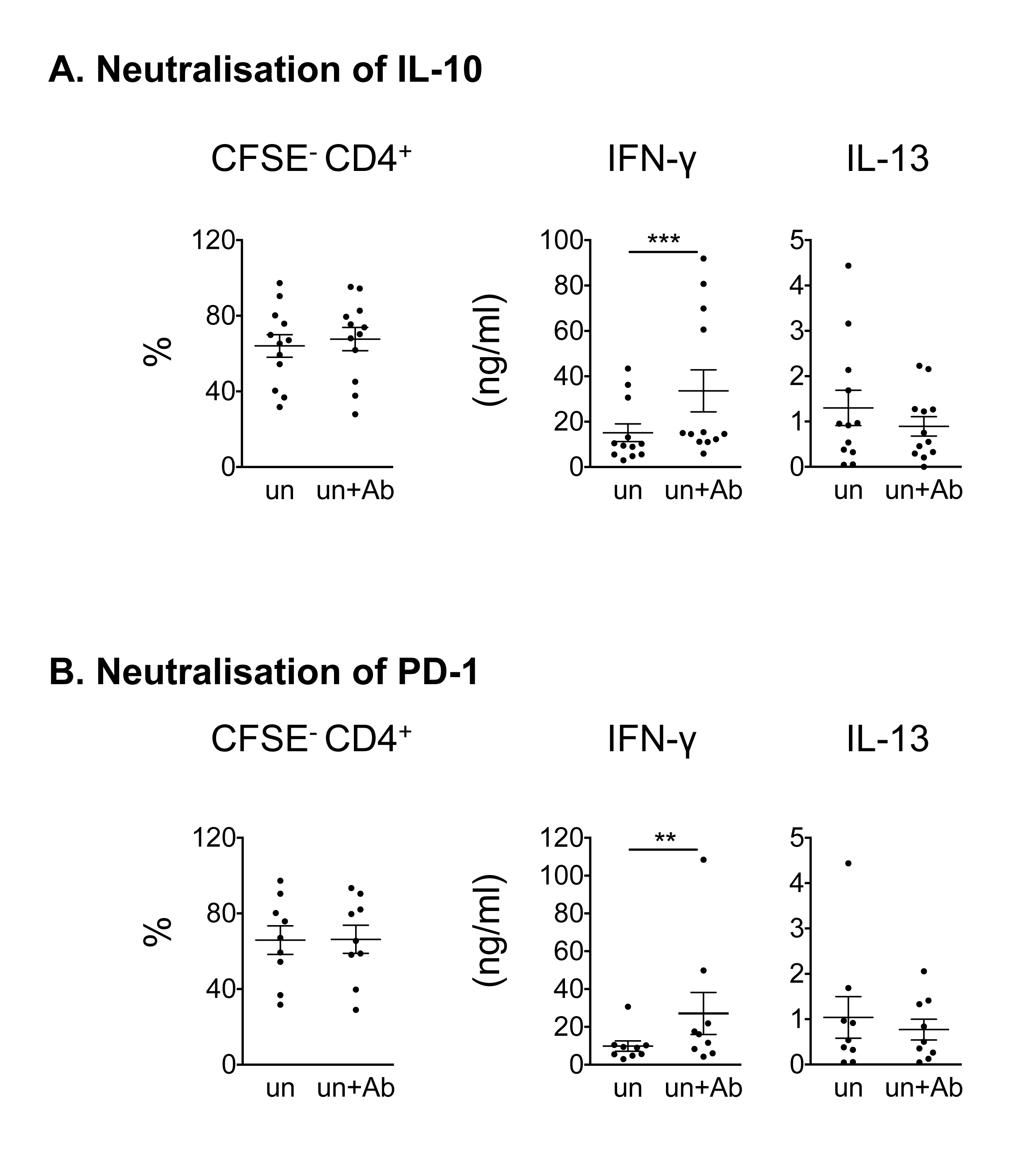

Supplement: Figure S4 — Neutralisation of IL-10 in cocultures with unstimulated monocytes. 5×10^5^ CFSE-labelled CD4+ T cells were incubated for 5 days with 1×10^5^ unstimulated monocytes +/− neutralizing antibodies (Ab) for A) IL-10 (pooled data from 4 experiments; n = 12) or B) PD-1 (pooled data from 3 experiments; n = 9). Proliferation (measured as CFSE dilution) of CD4+ T cells was measured by flow cytometry. Cytokine production was measured in the culture supernatant by ELISA. Horizontal dashed line indicates the limit of detection of the assay. All data are represented as mean ± SEM. P values were calculated using the Wilcoxon signed-rank test. ** p<0.01, *** p<0.001. (TIF) [file pntd.0003206.s004.tif]

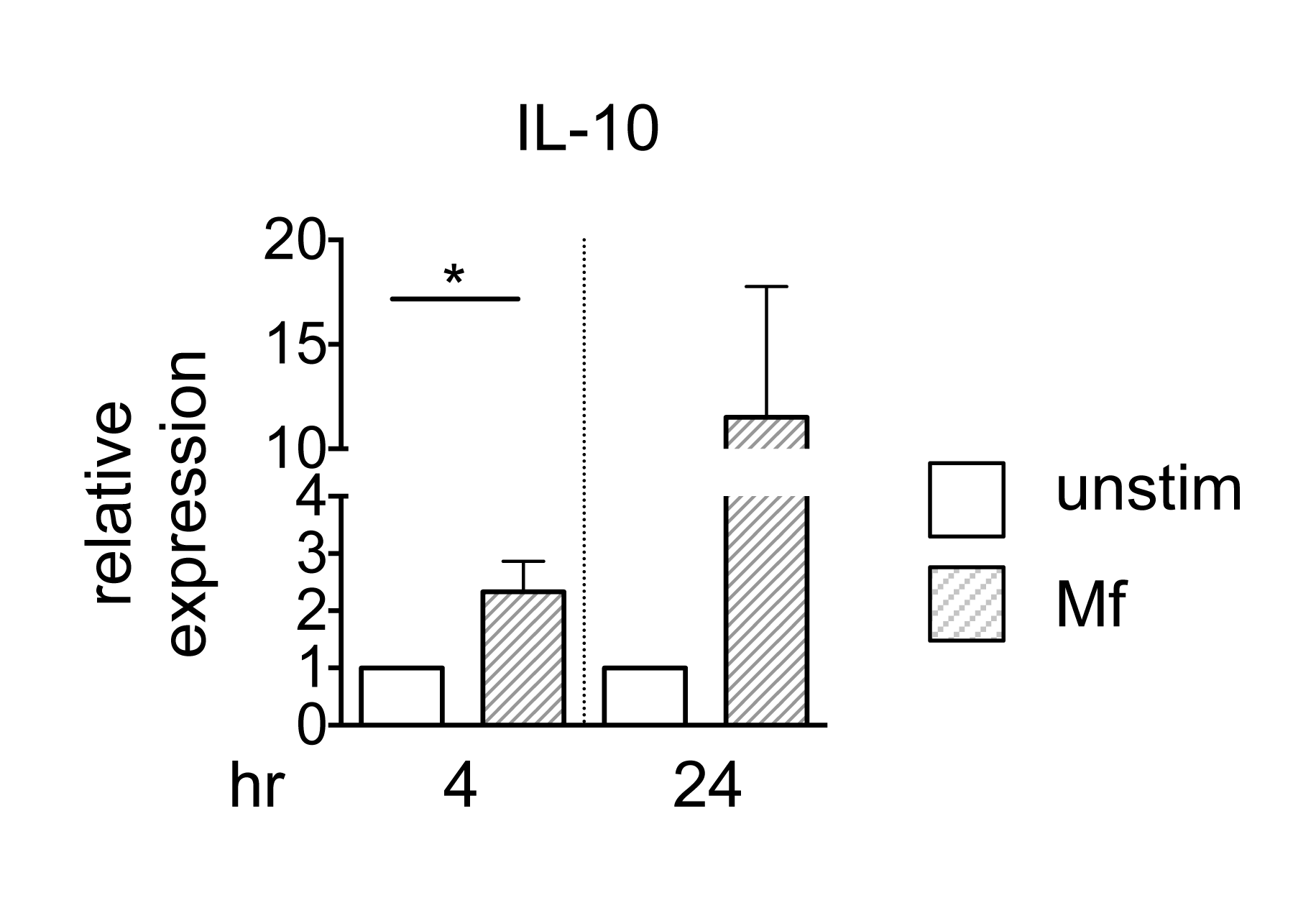

Supplement: Figure S5 — B. malayi Mf lysate-stimulated monocytes express IL-10 mRNA at the time of coculture. Monocytes were stimulated for 4 h or 24 h with B. malayi Mf lysate. mRNA expression was determined using RT-PCR (pooled data from 3 experiments; n = 9). All data are represented as mean ± SEM. P values were calculated using the Wilcoxon signed-rank test. * p<0.05. (TIF) [file pntd.0003206.s005.tif]

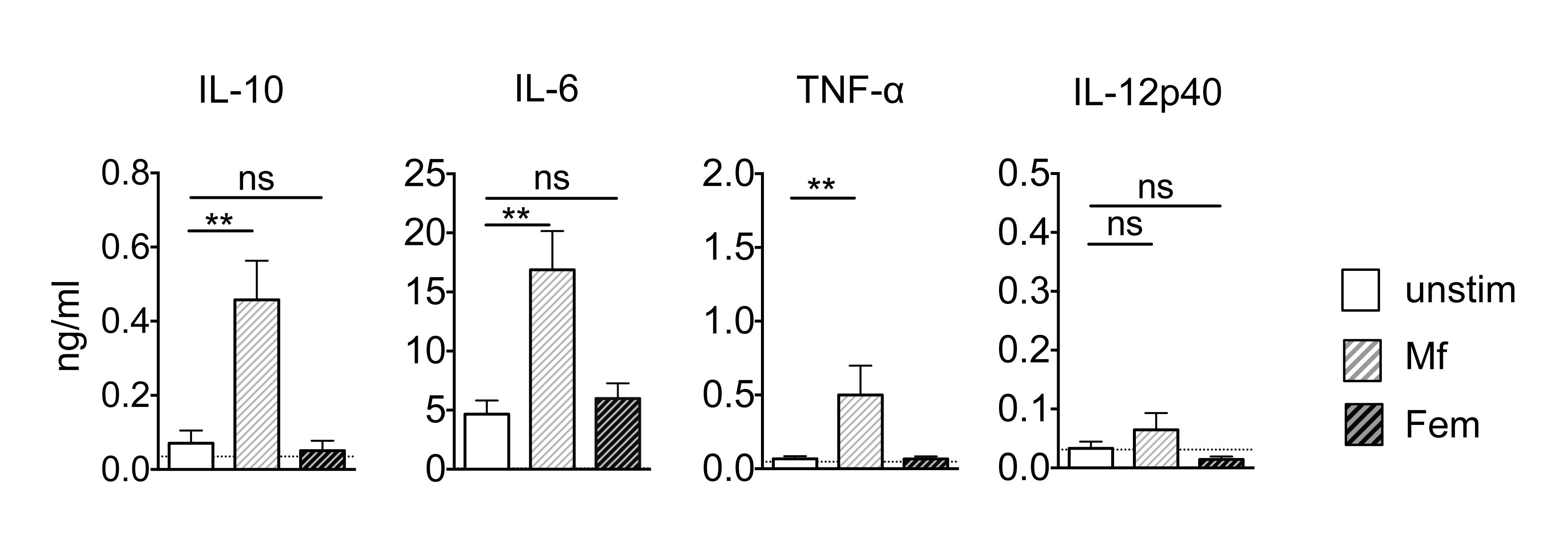

Supplement: Figure S6 — Mf lysate but not Fem lysate induces cytokine production in human monocytes. Human monocytes were stimulated for 24 h with 20 µg/ml microfilarial (Mf) or female (Fem) lysate and cytokines were assessed in the supernatant (pooled data from 4–8 experiments; n = 12–24). Data are represented as mean ± SEM. P values were calculated using the Wilcoxon signed-rank test. ns, not significant. ** p<0.005. (TIF) [file pntd.0003206.s006.tif]
