# Supplementary material for: Brugia malayi Microfilariae Induce a Regulatory Monocyte/Macrophage Phenotype That Suppresses Innate and Adaptive Immune Responses
Source: PLoS Negl Trop Dis. 2014 Oct 2;8(10):e3206. doi: 10.1371/journal.pntd.0003206 (PMC4183501; doi:10.1371/journal.pntd.0003206)
Supplement: Table S1 — Primer pair sequences used for real-time PCR. (DOCX) [file pntd.0003206.s007.docx]

| **Target** | **Accession number** |  | **Sequence** |
| --- | --- | --- | --- |
| *β2-microglobulin* | NM_004048 | Forward | 5'-TGC TGT CTC CAT GTT TGA TGT ATC T-3' |
|  |  | Reverse | 5'-TCT CTG CTC CCC ACC TCT AAG T-3' |
| *CCL18* | NM_002988 | Forward | 5'-TGT GCT GAC CCC AAT AAG AA-3' |
|  |  | Reverse | 5'-GGC ATA GCCA GAT GGG ACT CT-3' |
| *IL-6* | NM_000600 | Forward | 5'-ATG CAA TAA CCA CCC CTG AC-3' |
|  |  | Reverse | 5'-GAG GTG CCC ATG CTA CAT TT-3' |
| *IL-8* | NM_000584 | Forward | 5'-TAG CAA AAT TGA GGC CAA GG-3' |
|  |  | Reverse | 5'-AGC AGA CTA GGG TTG CCA GA-3' |
| *IL-10* | NM_000572 | Forward | 5'-AAG CCT GAC CAC GCT TTC TA-3' |
|  |  | Reverse | 5'-ATG AAG TGG TTG GGG AAT GA-3' |
| *IL-12p40* | NM_002187 | Forward | 5'-TCC ATC AGG ATC AGT CCC TA-3' |
|  |  | Reverse | 5'-GGT TTG CAT TGT CAG GTT TC-3' |
| *MRC-1* | NM_002438 | Forward | 5'-GGC GGT GAC CTC ACA AGT AT-3' |
|  |  | Reverse | 5'-ACG AAG CCA TTT GGT AAA CG-3' |
| *PD-L1* | NM_014143 | Forward | 5'-TGA TAC ACA TTT GGA GGA GAC G-3' |
|  |  | Reverse | 5'-CCC TCA GGC ATT TGA AAG TAT C-3' |
| *PD-L2* | NM_025239 | Forward | 5'-AGG CCT TTG ATA ATT GGC ACT A-3' |
|  |  | Reverse | 5'-CCC AAA TTT TGC TCA GTT AAG G-3' |
| *TNF-a* | NM_000594 | Forward | 5'-TCC TTC AGA CAC CCT CAA CC-3' |
|  |  | Reverse | 5'-AGG CCC CAG TTT GAA TTC TT-3' |

CCL, chemokine ligand; IL, interleukin; MRC, mannose receptor C; PD-L, programmed death-ligand; TNF, tumour necrosis factor.
